# Supplementary material for: Yes-Associated Protein 1 Is a Novel Calcium Sensing Receptor Target in Human Parathyroid Tumors
Source: Int J Mol Sci. 2021 Feb 18;22(4):2016. doi: 10.3390/ijms22042016 (PMC7922006; doi:10.3390/ijms22042016)
Supplement: Supplementary file 1 [file ijms-22-02016-s001.pdf]

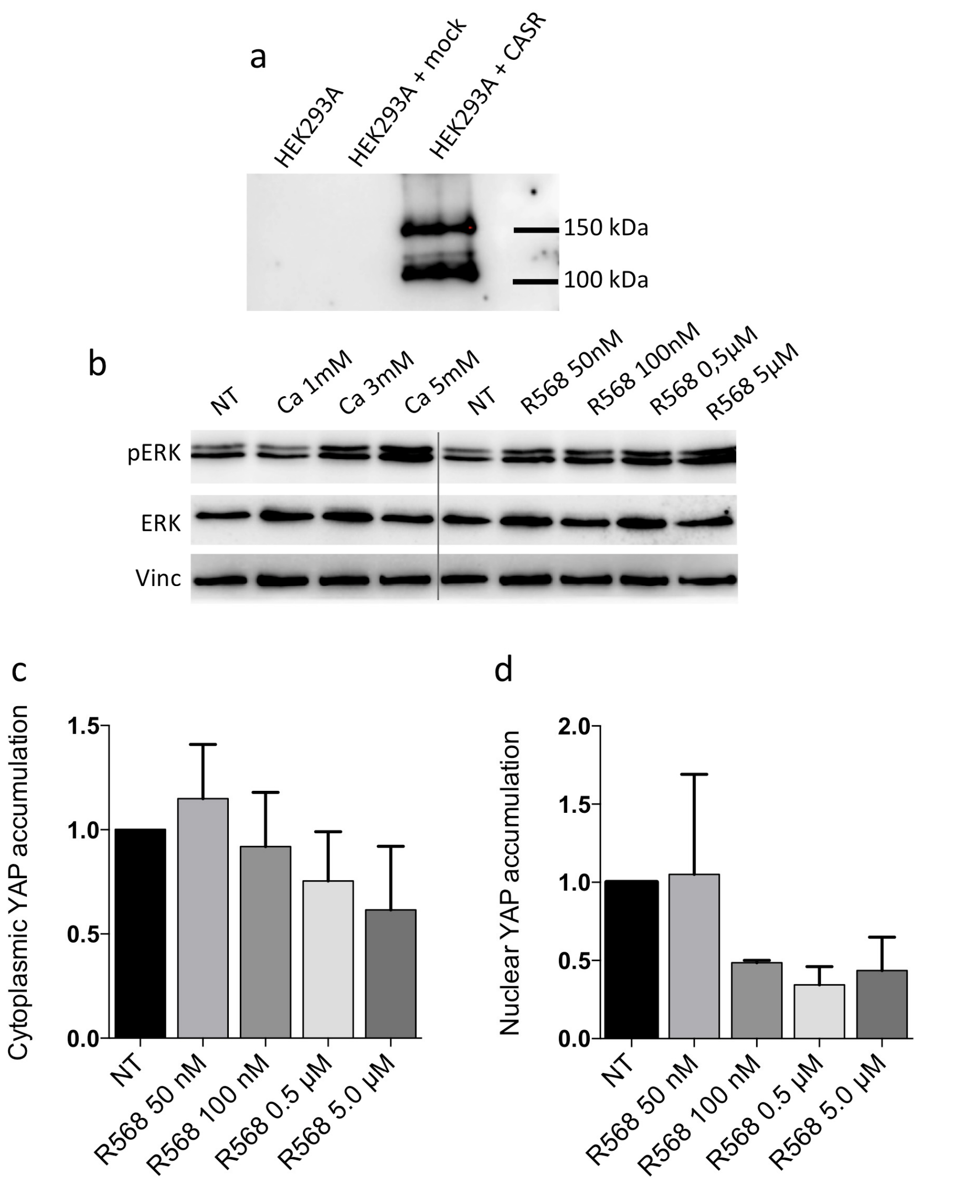

Supplementary Figure 1. (**a**) Detection of the calcium sensing receptor (CASR) protein after48 hours from transient transfection in HEK293A cells, with the expected double bands at 120 and 150 kDa. (**b**) Stimulatory effect of increasing $[Ca^{2+}]_o$ and of increasing concentrations of the CASR agonist R568 on phosphorylated ERK (pERK) and total ERK in HEK293A cells transiently transfected with CASR (CASR-HEK293A cells). (**c**) No effect of increasing concentrations of R568 on cytoplasmic YAP1 accumulation in HEK293A cells transfected with mock. (**d**) No effect of increasing concentrations of R568 on nuclear YAP1 accumulation in HEK293A cells transfected with mock
